# Shades of Serenity: Evaluating Orange Light as a Non-Pharmacological Intervention for Dementia

**DOI:** 10.1192/bjo.2026.11213

**Published:** 2026-06-30

**Authors:** Sneh Babhulkar, Sathyan Soundararajan, Zohreh Fouladi, Betsy Babu, Ugo Okafor

**Affiliations:** 1 NHS Greater Glasgow and Clyde, Glasgow, United Kingdom; 2 BCUHB, Wrexham, United Kingdom; 3 CWPT, Coventry, United Kingdom; 4 East London NHS Foundation Trust, London, United Kingdom; 5 Linn Dara, Cherry Orchard Hospital, Dublin, Ireland

## Abstract

**Aims:**

The purpose of this review was to methodically assess data from randomised controlled trials (RCTs) that looked at how orange-spectrum or circadian-relevant light interventions affected sleep, behaviour, and circadian outcomes in patients with dementia. Synthesising important findings and critically evaluating methodological quality using the Cochrane Risk of Bias 2 tool were secondary goals.

**Methods:**

A systematic research of PubMed was conducted from database inception to the most recent search date, using Medical Subject Headings and free-text terms related to dementia and light-based interventions. Only human RCTs were included. Outcomes of interest included sleep quality, agitation, circadian rhythm measures, and neuropsychiatric symptoms. Data were extracted into standardised tables, and methodological quality was assessed using the RoB 2 tool.

**Results:**

Five excellent RCTs satisfied the requirements for inclusion. People with moderate to severe dementia in residential care and community settings in the USA,UK and Europe were included in the study populations. Bright or circadian-effective lighting was the main intervention, though some studies also included less exposure to blue light in the evening. Agitation, sleep efficiency and circadian rhythm stability were the main results. The results showed mixed effects on agitation and behavioural symptoms, with only slight improvements in sleep and circadian outcomes. The overall risk of bias was rated as low to moderate, with blinding and selective outcome reporting being common issues.

**Conclusion:**

Circadian-oriented lighting interventions showed potential advantages for sleep and behavioural regulation, even though no trials specifically assessed orange-spectrum light. A promising non-pharmacological strategy for treating sleep and behavioural issues in dementia patients may be circadian-friendly lighting interventions. To improve the body of evidence and guide clinical recommendations, high-quality RCTs that specifically look at orange-spectrum lighting are needed.

